# Tailored Interventions to Improve Medication Adherence for Cardiovascular Diseases

**DOI:** 10.3389/fphar.2020.510339

**Published:** 2020-11-13

**Authors:** Hai-Yan Xu, Yong-Ju Yu, Qian-Hui Zhang, Hou-Yuan Hu, Min Li

**Affiliations:** ^1^ Department of Military Psychology, School of Psychology, Army Medical University, Chongqing, China; ^2^ Department of Social Work, School of Sociology and Law, Sichuan International Studies University, Chongqing, China; ^3^ Department of Foreign Languages, School of Basic Medicine, Army Medical University, Chongqing, China; ^4^ Department of Cardiology, Southwest Hospital, Army Medical University, Chongqing, China

**Keywords:** medication adherence, medication adherence interventions, tailored interventions, barriers to medication adherence, cardiovascular disease

## Abstract

Over the past half-century, medical research on cardiovascular disease (CVD) has achieved a great deal; however, medication adherence is unsatisfactory. Nearly 50% of patients do not follow prescriptions when taking medications, which limits the ability to maximize their therapeutic effects and results in adverse clinical outcomes and high healthcare costs. Furthermore, the effects of medication adherence interventions are disappointing, and tailored interventions have been proposed as an appropriate way to improve medication adherence. To rethink and reconstruct methods of improving medication adherence for CVD, the literature on tailored interventions for medication adherence focusing on CVD within the last 5 years is retrieved and reviewed. Focusing on identifying nonadherent patients, detecting barriers to medication adherence, delivering clinical interventions, and constructing theories, this article reviews the present state of tailored interventions for medication adherence in CVD and also rethinks the present difficulties and suggests avenues for future development.

## Introduction

During the past half-century, the medications used to treat cardiovascular disease (CVD) have improved greatly; however, patient adherence to medication recommendations is unsatisfactory, and nearly 50% of patients do not follow prescriptions when taking medications (Sabaté, 2003; Kronish and Ye, 2013). This situation limits the ability of maximizing therapeutic effects and results in adverse clinical outcomes and high healthcare costs (Bansilal et al., 2016). In its 2003 report, the WHO stated that “increasing the effectiveness of adherence interventions may have a far greater impact on the health of the population than any improvement in specific medical treatment” (Sabaté, 2003), and medication adherence has been considered the “next frontier in quality improvement” and an important part of cardiovascular outcomes research (Heidenreich, 2004). Therefore, improving medication adherence for CVD constitutes a significant part of clinical research.

### Medication Adherence and Barriers to Medication Adherence

The WHO defines adherence as “the extent to which a person’s behavior—taking medication, following a diet, and/or executing lifestyle changes, corresponds with agreed recommendations from a healthcare provider” (Sabaté, 2003), while the European consensus meeting defined adherence to medications as “the process by which patients take their medications as prescribed, composed of initiation, implementation, and discontinuation” (Vrijens et al., 2012). Generally, medication adherence means taking ≥80% of medication, poor/partial adherence means taking <80%, and medication nonadherence means taking <40% (Kronish and Ye, 2013; Bansilal et al., 2016; Baumgartner et al., 2018). Nonadherence can be classified into intentional vs. unintentional nonadherence according the barriers to medication adherence (Hugtenburg et al., 2013; Easthall and Barnett, 2017).

Barriers are the main reasons or impediments encountered by patients that hinder their ability to follow medication regimens (Eraker et al., 1984; Osterberg and Blaschke, 2005); thus, barriers may be the best targets of medication adherence interventions. For a long time, the expression of, approaches to, and roles of barriers were mainly referred to as *reasons* (Osterberg and Blaschke, 2005; Ho et al., 2009), *determinants* (Kardas et al., 2013), or *factors* (Ferdinand et al., 2017). Results from a bibliometric analysis of publications in medication adherence showed that it was not until 2008 that the word “barriers” increasingly replaced other words (Sweileh et al., 2019). Different barriers to medication adherence may exist for various diseases and medications as well as for different patients, even if they are diagnosed with the same disease or prescribed the same medication.

The classifications of and detailed factors associated with barriers to medication adherence may be different and numerous. The WHO has characterized five interacting dimensions: social and economic factors, healthcare teams and system-related factors, condition-related factors, therapy-related factors, and patient-related factors (Sabaté, 2003). In addition to this widely used classification system (Kardas et al., 2013; Ferdinand et al., 2017; Devine et al., 2018), there are other classifications, such as the 3-category patient, provider, and healthcare system factors, which possesses an interactive perspective (Osterberg and Blaschke, 2005), and the 2-category intentional and unintentional factors (Vrijens et al., 2012). Intentional barriers are mainly perceptual factors, such as illness perceptions and health beliefs, while unintentional barriers are usually practical factors, such as declining memory, complex regimens, and side effects of medication.

### Overview of Medication Adherence Interventions for Cardiovascular Disease

The effects of medication adherence interventions in the past half-century have been disappointing. There are very few randomized controlled trials (RCTs) reporting both improved adherence and clinical outcomes, and even the most effective interventions do not yield large improvements in CVD (Fuller et al., 2018). Even though there are new technological interventions, such as interventions delivered by mobile phones, the benefits of these mobile phone–delivered interventions for improving medication adherence for CVD are small, and some trials have found no beneficial effects from the intervention (Palmer et al., 2018).

There are two main approaches for improving medication adherence: single interventions and multicomponent interventions. Single interventions use a single method such as patient education, medication regimen management, convenient medication administration, reminders, psychotherapies, and so on, to improve patients’ medication adherence, while multicomponent interventions use multiple methods simultaneously. Many studies have concluded that multicomponent interventions are comparatively more effective than single interventions; thus, multicomponent interventions are usually recommended as a suitable way to improve medication adherence, given the concern that medication nonadherence is often multifactorial (Osterberg and Blaschke, 2005; Vonbank et al., 2017; Bosworth et al., 2018; Wang et al., 2019). However, multicomponent interventions also have disadvantages. They are complex, costly, and inconvenient in routine clinical practice and require multiple components (Osterberg and Blaschke, 2005; Kronish and Ye, 2013). Moreover, regardless of whether single interventions or multicomponent interventions are implemented, the current research usually lack focus, namely, the interventions targeted to neither nonadherent patients nor specific barriers. Thus, regardless of whether the patients were nonadherent and which types of barriers were present, patients received the same treatments. This one-size-fits-all approach may limit the effects of interventions and waste practitioner’s time and vigor.

Tailored interventions have been proposed as an appropriate way to improve medication adherence for many years (Sabaté, 2003; Baker et al., 2010; Baumgartner et al., 2018). They have been defined as “strategies to improve professional practice that are planned taking account of prospectively identified barriers to change” (Baker et al., 2010). With the key points of delivering interventions according to a patient’s specific barriers to medication adherence, tailored interventions involve an integrated process of identifying nonadherent patients, detecting barriers to medication adherence, and delivering potential solutions according to the patients’ barriers to medication adherence (Hugtenburg et al., 2013; Kronish and Ye, 2013; Xavier et al., 2016; Choudhry et al., 2018). However, tailored interventions necessitate a heavier workload and higher level techniques than most current interventions.

### Purpose and Methods

To date, no reviews of tailored interventions for medication adherence in CVD have been published. Due to the prevalence of poor medication adherence, disappointing effects of medication adherence interventions, and the hopeful potential of tailored interventions, methods to improve medication adherence must be reconsidered and reconstructed. There is a need to review the extant research on tailored interventions for medication adherence in CVD, with more in-depth and broad investigations of the present literature in terms of the main aspects of tailored interventions, and to learn actively from other disciplines (Armstrong and McAlister, 2016).

This study retrieved the literature from PubMed and MEDLINE databases published within the last 5 years (up to May 22, 2020) using the key words of *medication adherence, medication adherence intervention, cardiovascular diseases, tailored intervention, and barriers to medication adherence*. Reviews, RCTs, and observational studies focusing on the medication adherence, barriers to medication adherence, and tailored interventions of CVDs were retrieved. For those of relative importance, related literature were tracked, especially those trials of tailored interventions in CVD. Focusing on identifying nonadherent patients, detecting barriers to medication adherence, delivering clinical interventions, and constructing theories, this study reviews the present state of tailored interventions for medication adherence in CVD. It reconsiders the present difficulties and suggests avenues for future development. We hope this work will provide a reference for present and future interventions for medication adherence in CVD.

## Present State of Tailored Interventions for Medication Adherence in Cardiovascular Disease

### Identifying Nonadherent Patients

Identifying nonadherent patients who are candidates for tailored intervention is the first essential step in this process, and this requirement challenges the assessment of medication adherence, which has been a complex issue for nearly half a century. There are various methods used to assess medication adherence, including measurement of biologic markers in blood and/or urine, pill counts, pharmacy refills, electronic medication monitors, patient self-reports by questionnaires, and so on. However, none of these methods is considered a gold standard because each method has advantages and disadvantages (Osterberg and Blaschke, 2005). By contrast, objective methods are more popular than subjective methods. According to a systematic review and meta-analysis of 771 intervention trials, the largest effect sizes were reported by studies using electronic event monitoring for medication administration and pill count medication adherence measures (Conn and Ruppar, 2017). To balance the advantages and disadvantages of these methods, some authors have suggested using two or more methods to assess medication adherence, such as subjective methods along with objective methods (Lam and Fresco, 2015).

Because of their convenience and cost-effectiveness, self-report adherence questionnaires completed by patients are used widely to assess medication adherence in CVD (Uchmanowicz et al., 2019). However, they are easily confused with other questionnaires, such as questionnaires of barriers to medication adherence. According to the definitions of the WHO (Sabaté, 2003) and the European consensus meeting (Vrijens et al., 2012), the key point of medication adherence is the medication-taking behavior (Kronish and Ye, 2013). Therefore, it is rational to restrict the contents of questionnaires of medication adherence to medication-taking behaviors (e.g., *How often did you take your medications as the doctor prescribed?*), rather than barriers to medication adherence (e.g., *How often did you skip your medicines because/when* … or *Do you ever forget to take your medication*?).

In addition to the controversy surrounding the assessment methods, there is another issue in identifying nonadherent patients: the cutoff point of medication adherence. At present, the cutoff point of 80% of pills taken as prescribed is used widely to define adherence to cardiovascular medications (Kronish and Ye, 2013; Baumgartner et al., 2018), but this cutoff point is a convention only. Although it may be suitable for lipid-lowering medications for general coronary heart disease, it may not be suitable for anticoagulant therapy for postpercutaneous coronary intervention (PCI); the latter may need a higher level of medication adherence to ensure health. The optimal cutoff points for medication adherence for different CVDs remain poorly understood. Thus, cutoff points for nonadherence need to be confirmed individually for the different types of CVD.

### Detecting Barriers to Medication Adherence

In practical studies, barriers to medication adherence are usually presented as a series of single factors without a definite classification (Moss et al., 2014; Müller et al., 2015; Newman-Casey et al., 2015; Xavier et al., 2016). The common barriers to medication adherence for CVD include unaffordable costs, lack of belief in the necessity of medication, side effects of medication or medication concerns, complex regimens, forgetfulness, lack of family or social support, depression, lack of disease knowledge, and inconvenient access to medications or care (Choudhry et al., 2011; Nair et al., 2011; Müller et al., 2015; Zullig et al., 2015; Banerjee et al., 2016; Crawshaw et al., 2016; Xavier et al., 2016; Wang et al., 2019). There are some uncommon barriers that are reported relatively less often such as asymptomatic diseases (Devine et al., 2018), a poor provider–patient relationship, lack of understanding of the benefits of medication, prioritization issues or a busy lifestyle, disruptions to daily routines, and stigma or social embarrassment (Quach et al., 2009; Gellad et al., 2011; Nair et al., 2011; Farsaei et al., 2014; Müller et al., 2015; Newman-Casey et al., 2015; Zullig et al., 2015; Campbell et al., 2016; Sweileh, 2018; Venditti et al., 2018; Federman et al., 2019; Sweileh et al., 2019). For CVD, there are several diseases or conditions that may be symptomless such as hypertension, hypercholesterolemia, and even coronary heart diseases in some cases. Patients with asymptomatic diseases are more likely to be nonadherent.

Self-report tools are available for barrier information; thus, developing self-report tools for barriers to medication adherence is essential for tailored interventions. Perhaps, it is difficult to develop scales for barriers of CVD that strictly obey psychometrics, which require constructing theories and reliability and validity testing; thus, there are only a few scales for barriers that have reported both reliability and validity dates, such as the Identification of Medication Adherence Barriers Questionnaire (IMAB-Q) (Brown et al., 2017) and the Adherence Barriers Questionnaire (ABQ) (Müller et al., 2015). Validity testing is essential for barrier scales and usually accomplished by testing the barrier scales’ predictive value to medication adherence and clinical outcomes. Furthermore, scales for barriers developed by psychometric approaches usually have many items. Their measurement results are usually complex and require further explanation when presented to medical staff; therefore, they may be inconvenient when used in routine practice. Some studies have developed and used barrier checklists that contain the main barriers based on literature reviews and/or surveys without reporting theories construction and reliability and validity testing (Xavier et al., 2016; Lauffenburger et al., 2020). At present, there is no evidence regarding which tool development method is more suitable for research on barriers to medication adherence, especially for tailored interventions.

In the present research on tailored interventions to enhance medication adherence for CVD, open-ended questions (Choudhry et al., 2016), semistructured interviews (van der Laan et al., 2017), checklists (Hilbink et al., 2016; Xavier et al., 2016; Tahkola et al., 2018), barrier profiles (Hilbink et al., 2016), and scales (Nguyen et al., 2016) have been used as methods to identify barriers with different components. It is clear that these barriers will vary when assessed by different methods and components, and the results will lack comparability among different research studies. Therefore, it is necessary to evaluate the effects of different methods and identify common components of these barriers across different diseases, medicines, and patients. In addition, some studies have found that barriers to medication adherence include multiple factors (Lauffenburger et al., 2020), and there may be more potential tasks. Whether multiple factors have equal importance when affecting medication adherence and clinical outcomes, whether a core barrier is present and has high importance for the patient, and how to identify the core barrier in the process of tailored interventions remain unclear. More research is needed to answer these questions.

### Delivering Clinical Interventions

According to different classifications of nonadherence behaviors and barriers to medication adherence, tailored interventions may be designed with different projects (Hugtenburg et al., 2013; Kronish and Ye, 2013; Easthall and Barnett, 2017). For example, patients with intentional nonadherence mainly need behavior change techniques such as motivational interviewing and health coaching, while patients with unintentional nonadherence mainly need direct interventions such as reminder services, simpler regimens, and consultation with providers.

In clinical trials, tailored interventions of medication adherence in CVD are displayed with various forms (Table 1). Alfian et al. designed an adherence intervention wheel as supportive material for pharmacists to help patients with type 2 diabetes who were nonadherent to antihypertensive drugs in Indonesia (Alfian et al., 2020). This wheel contained four kinds of barriers and 15 specific barriers with one or more interventions. Hilbink et al. designed a graphic barrier profile that contained eight potential adherence barriers and a manual to identify barriers and determine interventions for patients starting cardiovascular or oral blood glucose–lowering medication (Hilbink et al., 2016). Tahkola et al. designed a 9-item checklist for the patient to complete with the treating physician to determine barriers and support tailored interventions (Tahkola et al., 2018). In the multicenter study by Xavier et al., trained community health workers used a list of possible medication and lifestyle barriers and conducted unstructured discussions with patients after acute coronary syndrome to determine strategies to overcome the barriers, thereby providing personalized interventions for each patient (Xavier et al., 2016). After 1 year of follow-up, these interventions improved adherence to drugs and healthy lifestyles and resulted in improvements in clinical risk markers. Choudhry et al. trained clinical pharmacists to use tailored multicomponent interventions by telephone to enhance medication adherence for patients with hyperlipidemia, hypertension, and diabetes (Choudhry et al., 2016; Choudhry et al., 2018). In this intervention, the clinical pharmacist used a brief negotiated interview to confirm the patient’s treatment regimen, identify barriers to adherence or other factors that may contribute to poor disease control, discuss the patient’s readiness to modify behaviors, and work with the patient to develop a shared plan to improve adherence and disease control. After 1 year of follow-up, this intervention resulted in a statistically significant increase in medication adherence but not clinical outcomes. Nguyen et al. proposed a measurement-guided medication management approach (Nguyen et al., 2016). They used the Medication Adherence Questionnaire (MAQ) to identify nonadherent patients who recently begun taking cardiovascular or oral hypoglycemic medication and the Beliefs about Medicines Questionnaire-Specific (BMQ-S) and the Brief Illness Perception Questionnaire (BIPQ) to identify potential barriers; they also designed a tool kit containing reminders, cognitive education, behavioral counseling, social support, and multifaceted interventions. There was a statistically significant improvement in adherence in the intervention group compared with the control group at 6 months. In the cardiovascular medication nonadherence tailored intervention (CATI), trained community pharmacies delivered patient-tailored interventions to enhance adherence to antihypertensive medication and to reduce cost (Bosmans et al., 2019; van der Laan et al., 2019). They developed a tailored intervention guide that contained five intervention modules, and each module included three components. However, this intervention did not improve medication adherence or result in cost-effectiveness.

**TABLE 1:** Clinical tailored interventions for medication adherence for cardiovascular disease (design and implementation).

Author	Participants	Identifying nonadherence	Assessment tool for medication adherence	Barriers to medication adherence	Interveners	Special tools for intervention	Outcomes	Follow-up time	Results
Alfian et al., 2020	Patients with type 2 diabetes who are nonadherent to antihypertensive drugs in Indonesia	Yes	Medication Adherence Report Scale (MARS)	4 kinds and 15 barriers	Pharmacists	Adherence intervention wheel	Medication adherence, BP level, medication beliefs, and process evaluation	3 months	Did not find report
Hilbink et al., 2016	Patients who started cardiovascular or oral blood glucose–lowering medication in the Netherlands	No	Probabilistic Medication Adherence Scale (ProMAS)	8 potential adherence barriers	Pharmacists	Graphic barrier profile	Medication adherence, nonadherence risk, predictive values of the baseline questionnaire in relation to medication adherence, barriers, and facilitators in the implementation of the tool	8 months	Did not find report
Tahkola et al., 2018	Hypertensive patients who were prescribed antihypertensive medication for the first time in Finland	No	No	3 kinds	Physicians	9-item checklist with hypertension knowledge, motivation, behavioral skills, and agreement	Ratio of setting an adequate BP target; ratio of remembering the adequate BP target; ratio of agreement at the next follow-up appointment	No	Increase in the ratio of setting a BP target; remembering the adequate BP target and agreement at the next follow-up appointment
Xavier et al., 2016	Patients with acute coronary syndrome in India	No	Composite medication adherence scale	7 barriers to drug adherence and 3 barriers to making adequate lifestyle changes	Community health workers	List of possible barriers to drugs and lifestyles	Medication adherence, lifestyle factors, and clinical risk markers	1 year	Statistically significant increase in overall drug adherence, smoking cessation, healthy diet, physical activity, and better clinical risk markers, except for diastolic BP and heart rate
Choudhry et al., 2018	Patients with hyperlipidemia, hypertension, and diabetes in the United States	Yes	Medication adherence from pharmacy claims data	6 categories for barriers by a semistructured interview	Pharmacists	Telephone consultations developed by using brief negotiated interviewing	Medication adherence from pharmacy claims data, disease control and healthcare resource use from claim data	1 year	Statistically significant increase in medication adherence but did not change clinical outcomes
Nguyen et al., 2016	Patients recently initiated on a cardiovascular or oral hypoglycemic medication in Australia	Yes	Medication Adherence Questionnaire (MAQ)	Medicine beliefs and illness perception	Pharmacists	Tool kit containing several strategies based on measurement of medicine beliefs and illness perception	Medication adherence	6 months	Statistically significant increase in medication adherence
Bosman et al., 2019	Patients treated with antihypertensive medication in the Netherlands	Yes	Pharmacy dispensing data (proportion of days covered), and Medication Adherence Report Scale (MARS-5)	A semistructured interview guide called the quick barrier scan (QBS) was used to explore patients’ barriers to medication adherence	Pharmacists	5 intervention modules from the tailored intervention guide (TIG); each module consists of 3 components	Medication adherence, beliefs about medicines, (BMQ concern and necessity scales), quality-adjusted life-years (QALYs), and costs	9 months	No significant differences in costs or effects between the intervention program and usual care

BP, blood pressure.

### Theoretical Construction

The importance of application of theory-driven, evidence-based models was highlighted in the research on medication adherence. Behavior change theories that contain a series of health psychology theories have been utilized to develop effective interventions, predict medication adherence, and identify barriers to medication adherence. Stavri and Michie advocated the development of a hierarchical classification system of behavior change techniques (Stavri and Michie, 2012). The system was a construction of theoretical frameworks (e.g., sociocognitive theory, self-regulation theory, and social support theory), within which a range of subordinate models (e.g., health belief model, theory of planned behavior, and self-regulation model) and individual components (e.g., perceived barriers, perceived benefits, treatment beliefs, and medication concerns) were included. This hierarchical classification system contained most of the main behavior change theories used in medication adherence. A systematic review by Holmes et al. found that the synthesis of studies highlighted the significance of self-efficacy, perceived barriers, perceived susceptibility, necessity beliefs, and medication concerns as predictors of medication adherence (Holmes et al., 2014).

Several behavior change models have been utilized to develop tailored interventions for medication adherence for CVD. The information–motivation–behavioral (IMB) skills model considered that behavior changes in medication adherence need adherence-related information, motivation (personal and social), and behavioral skills. This model was widely used in tailored interventions for HIV and has recently been used to assess barriers to medication adherence in patients with hypertension and diabetes (Jacobs et al., 2014; Nelson et al., 2018; Tahkola et al., 2018). The capability, opportunity, motivations-behavior (COM-B) model was developed as a behavior change wheel to explain the essential elements of behavioral change and was used to explore the determinants and barriers of medication adherence (Easthall and Barnett, 2017; Heneghan et al., 2020). Based on the COM-B model, Brown et al. developed the IMAB-Q, which focused on patients’ prescribed medicines for the prevention of CVD (Brown et al., 2017). The theoretical domains framework (TDF) was another integrative theoretical model, with 14 domains and 84 components (Cane et al., 2012), and Allemann et al. matched adherence interventions to patient determinants within 11 domains of the TDF (Allemann et al., 2016). Presseau et al. used the TDF and another behavior change theory, namely, the health action process approach (HAPA), to identify determinants of medication adherence following myocardial infarction (Presseau et al., 2017).

Many existing clinical tailored interventions did not report their particular theoretical construction. Among those with theoretical construction, behavior change theories are the main guides used for tailored interventions, with the following takeaways: first, comparatively, the IBM skills that have been used for patients with HIV, hypertension, and diabetes are more concise and can be understood and performed in clinical practice (Jacobs et al., 2014; Nelson et al., 2018; Tahkola et al., 2018), while some theories containing terminology from the original disciplines may need further modification and simplification for adaptation to the research on medication adherence and the rapid pace of clinical practice. Second, existing theoretical constructions of tailored interventions mainly focus on barriers to medication adherence. Because tailored interventions depend on the barriers identified, the method of classifying and detecting barriers is the main difference among tailored interventions. Third, comparing those barriers and determinants, research clearly reporting a particular theoretical construction placed more emphasis on patients’ internal factors to improve medicine-taking behavior, while clinical interventions without clearly reporting particular theories also emphasized patients’ external factors to overcoming barriers to medication adherence.

## Rethinks and Suggestions

It is important to acknowledge the prevalence of poor medication adherence and the disappointing effects of medication adherence interventions for CVD. Medication nonadherence is a complex issue involving many factors, and this further complicates the research on medication adherence interventions. Considerable effort has been undertaken to understand this problem, and great achievements have been made. Based on the findings of our predecessors, the following perspectives are presented in an attempt to rethink the present difficulties and propose avenues for future development.

### Redefining Tailored Interventions

Medication nonadherence is a complex problem for patients, providers, healthcare systems, and society as a whole. Tailored interventions for medication adherence for CVD are also complex procedures that involve referring to recruiting patients, identifying barriers, delivering interventions, and constructing theories. Therefore, it is very important to consider tailored interventions as a systematic research area, rather than an isolated clinical intervention. Thus, research on identifying nonadherent patients, detecting barriers to medication adherence, and determining and delivering potential solutions are absolutely necessary parts of tailored interventions. Overall, tailored intervention research includes identifying nonadherent patients, which involves developing appropriate assessment tools, identifying scientific cutoff point for medication adherence, detecting nonadherent patients as eligible participants, etc.; detecting barriers, which includes developing concise and practical tools for barriers to medication adherence, confirming the number and weight of barriers (single barrier or multiple factors, primary and secondary barrier), exploring the patient characteristics associated with barriers, identifying the main common barriers to medication adherence for different CVD, etc.; and determining and delivering potential solutions, which includes exploring tailored strategies, designing and implementing intervention, etc. Specifically, research on tailored interventions does not need to include all of the abovementioned parts but needs to consider all of the above parts at different research stages. For example, when recruiting participants, a tailored intervention needs to evaluate the tool used to assess medication adherence, the appropriate cutoff point for medication adherence, the eligibility of nonadherent patients as participants, and so on.

Considering the present research and clinical practice of managing CVD comprehensively, it is necessary to consider tailored interventions as continuous procedures. This requires two aspects. First, it is essential to consider tailored interventions as continuous procedures with three necessary steps: identifying nonadherent patients, detecting barriers to medication adherence, and determining and delivering potential solutions. This mainly directs at the first step because in the present research, the majority of studies on medication adherence (Allemann et al., 2017) and a considerable proportion of tailored interventions for CVD (Table 1) did not identify nonadherent patients as participants. Considering tailored interventions as a continuous procedure with these three steps helps avoiding this omission. Second, it is essential to consider the three steps of tailored interventions as dynamic and continuous procedures because medication nonadherence usually occurs gradually over time, and the barriers to medication adherence also may change at different times after the initiation of treatment. This is especially important for the assessment of barriers to medication adherence since most tailored interventions identified barriers only at the beginning of intervention and never assessed them again during the follow-up period; it is unknown whether the previous barriers had been overcome or new barriers to medication adherence had appeared. Dynamic and continuous evaluation of barriers to medication is needed to assess medication adherence and tailoring interventions.

It is necessary to consider tailored interventions for CVD as comprehensive interventions for the following reasons. First, in clinical research, tailored interventions are implemented to overcome barriers to medication adherence, while in behavior change theories, tailored interventions involve a process of changes in medicine-taking behavior from nonadherent behavior to adherent behavior. These two viewpoints are not inconsistent, as they can be the dual characteristics of tailored interventions that reflect the complexity of tailored interventions—not only to overcome certain barriers to medication adherence but also to change behavior concerning several factors. Therefore, tailored interventions may be defined as limited multicomponent interventions tailored to identified barriers: because behavior change is difficult and refers to several factors, tailored intervention with one component is not enough, and multicomponent interventions are needed. In addition, because interventions are tailored to barriers, each intervention is targeted to a specific barrier, the number of interventions is limited for each patient. Second, tailored intervention is not the sole intervention to improve medication adherence; it is not enough to deliver tailored interventions alone. Effective interventions such as health education, simplifying prescriptions, and lowering cost need to be considered in clinical practice as routine interventions to improve medication adherence in CVD. If considering improving medication adherence as comprehensive interventions, tailored interventions are the secondary prevention measures after those routine interventions; tailored interventions are important parts for improving medication adherence.

### Uniting Psychologists

Medication nonadherence is an interdisciplinary problem that refers to several disciplines, such as pharmacology, physiology, clinical medicine, psychology, and so on. There are many psychological components involved in the process of tailored interventions for CVD, including barriers to medication adherence, developing tools for those barriers, techniques to change medication-taking behaviors, and constructing theories for intervention. Psychologists play important and even irreplaceable roles in several areas of tailored interventions.

First, it is necessary to include essential psychological contents in barrier tools. There are many psychological factors that influence medication adherence (Crawshaw et al., 2016), and the necessary concern frameworks have been considered the fundamental barriers to medication adherence (Foot et al., 2016). These main psychological factors need be integrated into the tools of barriers to medication adherence for CVD. Second, regardless of overcoming barriers to medication adherence or changing medicine-taking behavior from nonadherence to adherence, successful tailored interventions need qualified communication techniques that are essentially psychological intervention techniques. At present, motivational interviewing was the mostly used psychological intervention in tailored interventions (Hugtenburg et al., 2013; Choudhry et al., 2016; Bosworth et al., 2017). However, there are thousands of psychological interventions, and many are brief and effective and have potential to be used in tailored interventions for medication adherence. They need be integrated into tailored interventions to increase the choices of communication techniques, and this combination may be another direction of research on tailored interventions with the need for further modification and simplification. Third, in the long term, theoretical construction is essential for tailored interventions, and psychological factors are also essential for theoretical construction. However, most present research on theoretical construction focuses on too many details of psychological factors and is complex and lacks conciseness and applicability. These research need further modification and simplification to adapt these findings to clinical practice. In summary, tailored interventions for medication adherence in CVD call for psychologists’ professional knowledge and skills.

### Reflecting Clinical Characteristic

The final aim of tailored interventions for CVD is to improve medication adherence and clinical outcomes and to lower costs. Thus, medication adherence, clinical outcomes, and costs should all be considered the outcomes of tailored interventions. At present, medication adherence is regarded as the outcome in nearly all clinical tailored interventions, but clinical outcomes and costs are seldom included. They need to be considered. In particular, clinical outcomes have been considered standards of high-quality medication adherence interventions (Nieuwlaat et al., 2014). For different CVDs, there may be different clinical outcomes. Major adverse cardiovascular events (MACEs), which include all-cause death, myocardial infarction, stroke, and coronary revascularization, have been used as outcomes of medication adherence interventions in many RCTs. They may be used as common outcomes of tailored interventions for CVD. However, since the clinical appearance of adverse outcomes requires a long follow-up time of clinical tailored interventions for CVD, the present follow-up times are usually within 1 year; it may be difficult to observe significant clinical outcomes, unless longer follow-up times are implemented.

There is a tendency that the majority of existing tailored interventions for CVD are delivered by pharmacists (5 of 7 in Table 1), and this trend is similar for most other diseases. In some countries, pharmacists have much more contact with patients, while in China, physicians and nurses are the main providers contacting with patients. Moreover, regardless of which place, it is physicians who prescribe medicines to patients; therefore, it would have been physicians’ responsibility to identify and intervene nonadherence in clinical routine (Ho et al., 2009; Armstrong and McAlister, 2016), or at least physicians should be the main implementers for interventions. However, clinical practice is very busy; thus, providers, especially physicians, may have little time and energy to address nonadherence to medication. If this is the reason for the absence of physicians and nurses in interventions for medication adherence, it is researchers’ responsibility to develop brief and effective interventions that can be seamlessly implemented into the busy clinical practice. Furthermore, tailored interventions obviously require a heavier workload and more complex techniques than the usual interventions for medication adherence. At the first glance, they are complex and expensive. To overcome these disadvantages and disseminate them in clinical practice, it is necessary to design brief protocols for tailored interventions. In summary, researchers always need to consider the costs of time, effort, expenditure, and technique at every step of tailored interventions and provide brief and effective protocols for tailored interventions via trial and error.

## Conclusion

Due to the complexity of medication adherence, just like the treatment for CVD, becomes more elaborate and targeted, interventions for medication adherence should also become tailored and individualized. Tailored interventions for medication adherence for CVD require further development. Overall, this is a systematic research area that includes research on identifying nonadherent patients, detecting barriers to medication adherence, and determining and delivering potential solutions. As a means of medication adherence intervention, tailored interventions should be dynamic and continuous procedures as well as comprehensive interventions. Psychologists play an important role in tailored interventions. Including clinical outcomes as outcome measures, emphasizing physicians’ roles as interveners, and simplifying protocols for interventions will allow for the adaptation of tailored interventions to clinical practice. This work may be difficult. However, just as an ancient Chinese poem said, *the way (to improve medication adherence for CVD) is long, but I (researchers) will always explore*.

## Author Contributions

H-YX, H-YH, and ML critically contributed to problem analysis and the construction of tailored intervention method, Y-JY and Q-HZ contributed to elucidate the introduction. The manuscript was amended based on comments from all authors. All authors read and approved the final version of the manuscript.

## Funding

This work was supported by the Humanities and Social Science Foundation of Army Military Medical University (2018XRW13) and the Youth Innovation Foundation of School of Psychology of Army Military Medical University (KY2018202).

## Conflict of Interest

The authors declare that the research was conducted in the absence of any commercial or financial relationships that could be construed as a potential conflict of interest.
